# Authentic Happiness at Work: Self- and Peer-Rated Orientations to Happiness, Work Satisfaction, and Stress Coping

**DOI:** 10.3389/fpsyg.2020.01931

**Published:** 2020-08-07

**Authors:** Nancy Tandler, Annette Krauss, René T. Proyer

**Affiliations:** ^1^Depratment of Psychology, Martin Luther University of Halle-Wittenberg, Halle (Saale), Germany; ^2^Interkantonale Hochschule für Heilpädagogik (HfH), Zurich, Switzerland; ^3^Department of Psychology, University of Zurich, Zurich, Switzerland

**Keywords:** coping, orientations to happiness, positive psychology, work satisfaction, work stress

## Abstract

The authentic happiness theory covers three basic orientations to happiness; namely, the life of pleasure (via hedonism); engagement (via flow-related experiences); and meaning (via eudaimonia). There is broad evidence for a positive relationship between these three orientations and indicators of positive psychological functioning in a variety of life domains. However, their contribution to well-being at work is understudied. The main aim of this study was testing the relationship between self- and peer-rated orientations to happiness, work related well-being (work satisfaction, work stress), and coping strategies. Further possible mediating effects of the coping strategies on the relationship between orientations to happiness and well-being at work were also examined. The sample consisted of 372 German-speaking Swiss adults (60.3% female), aged between 18 and 65 years (*M* = 38.9, *SD* = 10.8) with a minimum of 40% full-time employment. For 100 persons, peer-ratings of the orientations to happiness were available. Our results showed that the life of engagement and, to a lesser extent, the life of meaning are related to work satisfaction. The life of pleasure was associated with lower levels of reported work stress. Further, positive associations between self- and peer-rated orientations to happiness (particularly pleasure) and adaptive coping strategies with stress were also found. Mediation analyses showed that the effects of engagement in general and content-related work satisfaction were mediated mainly by control and negative coping, while the association between meaning and resigned work satisfaction was mediated by positive coping. Negative coping fully mediated the association between the pleasurable life and work stress. Overall, our results indicate that employees’ orientations to happiness are of importance for experiencing well-being at work.

## Introduction

Well-being at work is not only crucial for the overall well-being of the individual but also leads to better work performance (e.g., Judge et al., 2001; Zelenski et al., 2008; Cooper et al., 2019), higher levels of employee creativity and engagement (Bartels et al., 2019), and has been associated with lower rates of absenteeism at work (e.g., Wegge et al., 2007; Ybema et al., 2010). To effectively enhance work-related well-being and the ability to cope with work-related demands, approaches that help researchers to understand the mechanisms that add meaning to life and facilitate optimal functioning seem promising (Seligman and Csikszentmihalyi, 2000; Gable and Haidt, 2005). Accordingly, recent years have seen a growing interest in studying positive psychology in association with organizational behavior (see, for example, Luthans and Youssef, 2007). In an effort to narrow a gap in the literature, we examine three basic orientations to happiness at work and their association with work satisfaction and work stress. In particular, we will collect self- and other ratings in the orientations and see whether they relate to specific stress-coping strategies and could thus be seen as protective factors buffering against stress.

### Orientations to Happiness

Positive psychology is an “[…] umbrella term for the study of positive emotions, positive character traits, and enabling institutions” (Seligman et al., 2005, p. 410). There has been growing interest in the field of positive psychology over the past decade (Donaldson et al., 2011, 2015), and its main areas of interest are (a) *positive subjective experiences* such as happiness, (b) *positive individual traits* such as orientations to happiness, and (c) *positive institutions* such as schools and workplaces, etc. (Seligman and Csikszentmihalyi, 2000). Positive institutions should encourage the use of positive traits, which, in turn, leads to positive experiences (see e.g., Harzer and Ruch, 2013; Pang and Ruch, 2019). Hence, the application of models derived from positive psychology and applied to the workplace facilitates the study of the potential contributors to positive experiences at work, for example, the role of hedonic, eudaimonic, and flow-related personal resources.

In his *authentic happiness theory*, Seligman (2002) postulates three routes to happiness: the pleasant life, the engaged life, and the meaningful life. The pleasure orientation (akin to the notion of *hedonism*) suggests the maximization of pleasure and the minimization of pain as the main route to happiness. Already, Aristuppus argued that immediate sensory gratification is the main way to experience a fulfilled life (Watson, 1895). The engaged life route builds upon works on “flow” experiences (Csikszentmihalyi, 1990) for achieving a good life. During flow, people’s attention is focused on the task itself, and they are absorbed with the activity. Flow is different from sensual pleasure, and it is argued that it is rather non-emotional and non-conscious in the moment. The third route to happiness, the meaningful life, results from individuals’ using their signature strengths (i.e., morally positively valued traits; see Peterson and Seligman, 2004) to serve something larger than themselves (akin to the pursuit of *eudaimonia*). Being true to one’s inner self (*demon*), that is, identifying and cultivating one’s strengths and virtues and living in accordance with them in the service of greater goods, leads to happiness (Aristotle, 2000). Hence, two of the three orientations have long-standing roots in philosophy (hedonism and eudaimonia), while engaged life has its roots in psychology (flow experiences). Of course, hedonism and eudaimonia are also studied outside the framework of the authentic happiness model, namely, as facets of well-being, hedonic and eudaimonic well-being (e.g., Di Fabio and Kenny, 2018), and have led to the development of a new theoretical framework considering sustainable well-being in organizations (*Psychology of Sustainability*; Di Fabio, 2017).

Previous research on adults from 27 different nations has revealed that the orientations to happiness are related but remain distinct and positively correlated with life satisfaction and other indicators of positive psychological functioning (Peterson et al., 2005, 2007; Park et al., 2009). Numerically, engagement displayed the strongest and pleasure the weakest associations. Peterson et al. (2005) showed that those people with a “full life” (i.e., high expressions in all three orientations) are most satisfied with their lives, while those with low expressions in all three orientations (i.e., an “empty life”) are less satisfied; this notion has received support from a study on the perception of daily activities in terms of the three orientations and the importance of a balance across all three (Grimm et al., 2014). There is also evidence from placebo-controlled intervention studies that deliberate activities targeting the three orientations (either separately or jointly) are associated with an increase in (a) the orientations themselves, (b) happiness, and (c) an amelioration of depression (e.g., Giannopoulos and Vella-Brodrick, 2011; Gander et al., 2016, 2017, 2018).

### Orientations to Happiness and Well-Being at Work

The relationship between the orientations to happiness and work satisfaction as one dimension of well-being at work (Rothmann, 2008) has been less frequently studied than their association with (the facets of) subjective well-being (e.g., Peterson et al., 2005; Park et al., 2009). Nevertheless, there is robust evidence for positive contributions from the three orientations to one’s working life. For example, Hofmann et al. (2018) recently identified that the associations between the orientations to happiness and life satisfaction differ across occupations. Using a representative sample of the Swiss workforce (*N* = 1,140), they showed that people in more highly skilled occupations (defined as a function of the complexity and range of tasks and duties to be performed in an occupation) reported higher life satisfaction and a lower orientation to pleasure than those in less skilled occupations. In addition, the orientation to meaning was more closely related to life satisfaction in highly skilled than in less skilled occupations. The three orientations also relate to career adaptability (Johnston et al., 2013). Proyer et al. (2012) tested 221 career officers in the Swiss military and found that the engaged and meaningful life positively and robustly correlated with work satisfaction. Engagement showed a strong association with the satisfaction with content-related aspects of work, whereas the life of meaning also correlated with general work satisfaction. Furthermore, the orientations were also predictive of the subjective and objective indicators of *career success* (the latter operationalized by military rank).

Swart and Rothmann (2012) tested managers in the agricultural sector in South Africa (*N* = 507) and found that all orientations to happiness, but especially engagement, had an impact on work satisfaction. Furthermore, the relationship between the orientations to happiness and work satisfaction were substantiated using longitudinal data. Martínez-Martí and Ruch (2017) discovered an association between orientations to happiness and work satisfaction 1 year after a first measurement period in a representative sample of employees in Switzerland. They stated that, although engagement was a better predictor than pleasure or meaning, the simultaneous endorsement of all of the three orientations is the best predictor for work satisfaction (i.e., the “full life;” Peterson et al., 2005). Using a large sample of 1,134 Japanese working adults, Suzuki et al. (2015) established an association between the life of meaning and grit (the perseverance and passion for long-term goals). Although the latter has been discussed critically with respect to its distinctiveness (e.g., Credé et al., 2017; Vazsonyi et al., 2019), this may be seen as a hint for an interplay between the orientations to happiness and their role in goals that relate to perseverance. Finally, the pursuit of work-related activities is robustly associated with the engaged life (and the meaningful life; Ruch et al., 2010, 2014). Overall, a balance between the pleasurable, engaged, and meaningful life may help to achieve sustainable happiness. Given that adults typically spend a considerable portion of their time at work, testing the role of the three orientations to happiness for work-related outcomes and dealing with stress seem particularly important.

#### The Role of Work Stress

Positively dealing with work stress is an important contributor to well-being at work (Rothmann, 2008). We follow a transactional perspective on *stress* (e.g., Lazarus and Folkman, 1984a, b) and use De Bruin’s (2006) definition of work stress, namely, “[…] an uncomfortable state of psychological tensions that results from an appraisal that the perceived demands of the workplace exceeds the individual’s perceived resources to successfully meet the demands” (p. 68). Based on the assumption that orientations to happiness function as personal resources and contribute to lower levels of stress at work, Johnston et al. (2013) demonstrated that the orientations to happiness (especially engagement) positively correlated with career adaptability (i.e., individuals’ behaviors, competencies, and attitudes engaged “in fitting themselves into work that suits them;” Savickas, 2005, p. 45). Furthermore, pleasure and engagement were negatively correlated with work stress. We aim to replicate and extend the latter finding by taking the potential limitations of subjective data into account.

Most of the earlier studies on the orientations to happiness were based on self-reports alone (for an exception see, for example, Ruch et al., 2010). One limitation of this approach is that these ratings may be prone to distortions due to the answer styles (e.g., social desirability or extreme answering behavior; for an overview, see Furnham, 1986). Thus, a method bias (e.g., Campbell and Fiske, 1959) may have an impact on the findings (e.g., an overestimation of the true effects due to similar answering behavior in the measures used). Another limitation may be that self-ratings do not provide the full picture of the associations, as it has been shown that ratings by acquaintances contribute additional information (e.g., Connelly and Ones, 2010). Thus, the inclusion of ratings by knowledgeable others will provide a second source of data to help in testing the stability of the associations beyond mere self-reports. Earlier research has shown that self- and peer ratings in the orientations to happiness converge well (around 0.50; Ruch et al., 2010); this is within the approximate range that has been reported for trait measures in personality research [i.e., between 0.46 (neuroticism) and 0.62 (extraversion); Connolly et al., 2007]. Overall, we expect that self- and peer-rated orientations to happiness, and especially engagement, would be correlated with work satisfaction.

### Coping Strategies Mediating the Orientations to Happiness—Well-Being at Work Link

We derive our hypotheses from the transactional model of stress (Lazarus and Folkman, 1984a, b). Here, psychological stress is seen as a transaction between individual and environmental factors and is based on two basic forms of appraisal: primary and secondary appraisal. Primary appraisal refers to a person’s cognitive appraisal whether something of relevance to the individual’s well-being occurs, whereas secondary appraisal concerns coping options. These appraisals are determined by a number of personal variables such as motivational dispositions, goals, values, and generalized expectancies. In terms of the transactional model of stress (Lazarus and Folkman, 1984a, b), orientations to happiness may serve as personal resources of working people that help to explain the way people deal with work-related challenges and stressors. Orientations to happiness should facilitate the use of particular positive and negative coping strategies that, in turn, help to deal with challenges in terms of experiencing lower levels of stress in the workplace. In line with this theoretical framework, we aim to study the mediating effects of coping strategies for a better understanding of how the orientations may contribute to well-being at work.

Coping is defined as an individual’s habitual way of reacting to stressors by adopting certain strategies (Erdmann and Janke, 2008). *Positive* and *negative coping strategies* can be distinguished: Positive coping strategies (e.g., positive self-affirmation, relaxation) reduce the experiencing of stress, while negative or maladaptive coping strategies consist of behaviors that alleviate the feeling of stress briefly, but not in the long run (e.g., escape, social withdrawal, rumination). In line with Fredrickson’s (2004), *broaden and build theory of positive emotions*, we argue that the pursuit of the three orientations is linked to the elicitation of positive emotions. Referring to earlier work by, for example, Isen (e.g., Isen and Levin, 1972), Fredrickson argues that positive emotions serve as efficient antidotes for the lingering effects of (e.g., stress-induced) negative emotions. By broadening a person’s momentary thought–action repertoire, a positive emotion may buffer the effects of negative experiences. Positive emotions could, therefore, help broaden the scope of attention, cognition, and action and build physical, intellectual, and social resources. In line with these assumptions, research has shown that positive emotions are crucial facilitators of adaptive coping and adjustment to acute stress (Folkman, 1997; Folkman and Moskowitz, 2000). Within the transactional stress model (Lazarus and Folkman, 1984a, b), Fredrickson’s (2004) *broaden and build theory of positive emotions* can help to explain an individual’s *coping potential*. Coping potential refers to the appraisal processes and means an individual’s evaluation of the expectation for generating certain cognitive or behavioral operations that will positively influence a personally relevant encounter. In short, it is argued that the three pursuits may facilitate the usage of specific, positive strategies that help in ameliorating perceived stress and combat-related negative outcomes. In this sense, the orientations could well be seen as a resource that facilitates the usage of certain stress-reducing strategies or prevents maladaptive strategies from occurring and, ultimately, leads to lower levels of perceived stress. Consequently, we expect a positive relationship between the orientations to happiness and positive coping strategies and a negative association between the orientations and negative coping strategies.

To the best of our knowledge, there has been no study that examines the relationship between orientations to happiness and coping with stress. It is argued that the pursuit of the three orientations will be linked to specific types of coping strategies (referring to Erdmann and Janke’s, 2008). For example, those who pursue a hedonistic approach to happiness may distract themselves by conducting other activities that help them to relax. Those that pursue a life of engagement and experience flow have feelings of total concentration and absorption and an invigorating feeling of having things under control arises (Csikszentmihalyi, 1990). We, therefore, expect that engagement is linked to the coping strategies of control and distraction. Distraction in terms of Erdmann and Janke’s (2008) is a positive strategy that enables people to direct their attention to activities that are useful to divert oneself from a stressful situation and/or to engage with activities that are incompatible with the stressful situation. A life of meaning will be associated with the search for higher values in life wherein problems and difficulties are understood in a larger context. Individuals who pursue this orientation may have a particular propensity for the usage of coping strategies that help them to cognitively restructure current stressful incidents.

### Aims of the Study

The purpose of the present study is to test the associations of self-reported orientations to happiness with well-being at work (work satisfaction, work stress) and extend this by, additionally, analyzing the associations of peer ratings for the three orientations with facets of work-related well-being. We expect that the orientations to happiness, especially engagement, are positively associated with work-related well-being—in both the self- and peer ratings (H1). Second, we test the relation between self- and peer-rated orientations to happiness and coping strategies (H2). We expect that the self- and peer-rated orientations to happiness are positively related with positive coping and negatively with negative coping mechanisms. Third, we hypothesize that the relationship between orientations to happiness and well-being at work is mediated by the coping strategies (H3).

## Materials and Methods

### Participants

#### Sample 1: Self-Ratings

We collected data from *N* = 372 German-speaking working adults in Switzerland (60.2% female, 39.8% male) aged from 18 to 65 (*M* = 38.9, *SD* = 10.8). The only inclusion criterion was that they had to be employed with a minimum of 40% full-time employment (FTE = 42 h/week in Switzerland). The participants reported an average percentage of working hours per week of 89.8% (*SD* = 18.5; range, 40–100%). The majority of the participants held a university degree (72.6%), 25.3% completed secondary education, and 2.2% reported a level of education lower than elementary school.

#### Sample 2: Peer-Ratings

For 100 participants, peer ratings of the orientations to happiness were available from acquaintances (60.0% female, mean age = 40.0, *SD* = 11.0, range, 24–66 years). The majority of the acquaintances (66.0%) indicated that the rated person was their partner or spouse, while 16.0% indicated that the rated person was a friend. Most of them knew the rated person very well; on a scale from 1 = “hardly” to 7 = “excellent,” 51.0% indicated a “6,” and 38.0% a “7” level of acquaintance. The lowest rating was “4,” while the mean value was *M* = 6.2 (*SD* = 0.8).

### Procedure

Data were collected in an online survey hosted by an institution of higher education in the German-speaking part of Switzerland. The study was announced on internet forums, social networks, and via leaflets. Participants were not paid but received individual feedback on the study variables. They were informed that feedback was only given for the self-ratings but not for the ratings by knowledgeable others. The peer raters were also informed that their evaluation would not be shared with the target person. The peer ratings for the Orientations to Happiness scale (Peterson et al., 2005) were also collected online using an individualized link sent to the person. The study was conducted in line with institutional standards and recommendations. All procedures complied with the ethical guidelines of the local ethics committee at the institution of higher education where the study was conducted. Participation was voluntary, anonymity was ensured, and participants could end the survey at any time, without consequences. Consent was obtained by virtue of survey completion.

Collecting data in online studies has been criticized for several reasons (e.g., sample biases). However, research has shown that data collected online are comparable to those collected in conventional ways (Gosling et al., 2004). We designed and conducted our study according to the code of “good practice” in internet-delivered assessments (Coyne and Bartram, 2006). All data are available on the Open Science Framework: https://osf.io/mf5cj/.

### Measures

#### Orientations to Happiness

Orientations to happiness were assessed using the German version (Ruch et al., 2010) of the *Orientations to Happiness Scale* (OTH-self; Peterson et al., 2005). This consists of 18 items, and answers were given on a 5-point Likert scale (1 = very much unlike me, 5 = very much like me). The OTH scale is the standard measure for the assessment of the three orientations and frequently used in research across various contexts and cultures (Giannopoulos and Vella-Brodrick, 2011; Buschor et al., 2013; Johnston et al., 2013). The internal consistencies in this sample were satisfactory for research purposes, namely, α = 0.73 (life of pleasure; six items, e.g., “Life is too short to postpone the pleasures it can provide”), α = 0.68 (life of engagement; six items, e.g., “I am always very absorbed in what I do”), and α = 0.79 (life of meaning; six items, e.g., “My life serves a higher purpose”).

Participants in Sample 2 completed a *peer-report form of the Orientations to Happiness Scale* (OTH-peer; Ruch et al., 2010), which uses the same 18 items as the OTH-self, but rephrased to enable ratings by a third person. Ruch et al. (2010) reported convergence between 0.48 and 0.51 between self- and peer ratings. The internal consistency coefficients for the OTH-peer were comparable to those of the self-reports (i.e., α = 0.75 for the pleasurable, six items, e.g., “For her/him, life is too short to postpone the pleasures it can provide;” α = 0.64 for the engaged, six items, e.g., “He/She is always very absorbed in what he/she does;” and α = 0.80 for the meaningful life, six items, e.g., “His/her life serves a higher purpose”).

#### Satisfaction With Work

Work satisfaction, as a subjective experience of objective work situations, was assessed using the German version (van Dick et al., 2001) of the *Job Diagnostic Survey* (JDS; Hackman and Oldham, 1975). The total scale comprises 24 items. Three subscales of general work satisfaction (4 items, e.g., “In general, I am happy with my work”), content-related work satisfaction (15 items, e.g., “I perceive my work as very varied”), and resigned work satisfaction (4 items, e.g., “Even though my work is not ideal, it could be worse”) were built.

Participants had to respond on a 6-point Likert scale (1 = strongly disagree, 6 = strongly agree). The resigned work satisfaction subscale, however, was answered on a 7-point Likert scale (1 = practically never, 7 = practically always). The internal consistencies and items of the three types of work satisfaction were good (general work satisfaction: α = 0.88; content-related work satisfaction: α = 0.86; resigned work satisfaction: α = 0.81).

#### Work Stress

The *General Work Stress Scale* is a one-dimensional measure of the level of stress caused by work (De Bruin and Taylor, 2005). We used a German translation that was also used in Johnston et al. (2013). Individuals responded to nine items (e.g., “Do you become so stressed that you would resign?”) using a 5-point Likert type scale (1 = never, 5 = always). For this study, Cronbach’s alpha was 0.90.

#### Coping

Coping with stress was measured with the German-language questionnaire *Stressverarbeitungsfragebogen* (“stress coping questionnaire;” SVF-120; Erdmann and Janke, 2008). We used the standardized and adapted version assessing stressful situations at the *workplace* [using this instruction: “When I was affected, upset, or overbalanced by work (…)”]. The SVF-120 consists of 120 items, and answers are given on a 5-point Likert scale answer format (0 = not at all, 4 = very likely). Twenty different coping modes can be distinguished and subsumed into two broad categories, namely, positive and negative coping strategies (plus four equivocal coping modes, which are not of interest here). To choose an appropriate level of analysis, we followed the authors’ (Erdmann and Janke, 2008) suggestions to distinguish between negative and positive strategies. The positive strategies can further be separated into three subcategories, whereas this does not apply for the negative strategies. The first positive coping subcategory, *devaluation*, covers a cognitive method of coping and entails the minimization, self-aggrandizement by comparison with others, and denial of guilt coping modes. The second, *distraction*, is characterized by seeking distraction from strain by focusing on situations and states that are incompatible with stress. It entails four coping modes: distraction, substitute gratification, search for self-affirmation, and relaxation. *Control* is the third positive coping subcategory and entails the active control of stressors and reactions. The related coping modes are situation control, reaction control, and positive self-instructions. *Negative coping strategies* are escape, social withdrawal, rumination, resignation, self-pity, and self-blame. The SVF-120 is frequently used in German-speaking countries (e.g., Rammsayer et al., 2006; Brandhorst et al., 2016).

Internal consistency coefficients and item samples were as follows: devaluation [α = 0.86; e.g., “(…) I take it easier than other people.;” to deemphasize], distraction [α = 0.90; e.g., “(…) I try to distract myself somehow.;” distraction], control [α = 0.87; e.g., “(…) I tell myself that I can handle it.;” positive self-instruction], and negative coping (α = 0.96; e.g., “[…] I avoid people.;” social withdrawal). A more detailed description of all subscales of the SVF-120 are available in the electronic supplement (Supplementary Table S1)^1^.

### Statistical Analyses

**FIGURE 1:**
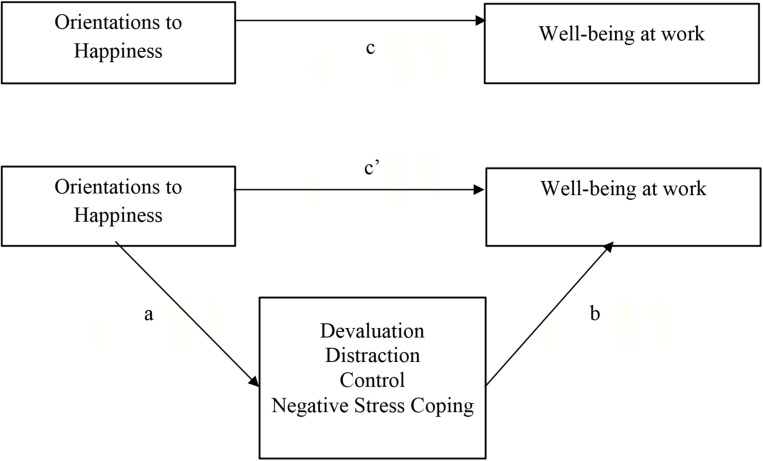
Parallel mediational model. Total (*c*), direct (*c’*) and indirect effects (*ab*) of the orientations to Happiness (Pleasure, Engagement, Meaning) on Well-being at work (work satisfaction, work stress) via the mediators Positive (Devaluation, Distraction, Control) and Negative Coping Strategies.

Following correlation analyses, we conducted hierarchical multiple regression analyses for each work satisfaction facet (general, content-related, and resigned; three separate analyses) and for work stress as criteria. We controlled for age and gender in Step 1 because these features are known to vary with facets of well-being (e.g., Peterson et al., 2005) and are considered to be “personal dispositions” in our theoretical stress model (Lazarus and Folkman, 1984a, b). Subsequently, we entered the three orientations to happiness as a predictor set in Step 2 to study their explained variance independently of age and gender. Additionally, we performed mediation analyses to test whether relations between orientations to happiness and types of work satisfaction and work stress are mediated by coping strategies. Figure 1 illustrates this mediation model. These analyses were performed using the PROCESS script (model 4) by Hayes (2013), which allows us to estimate total (*c*), direct (*c*’), and indirect effects (*ab*) in multiple parallel mediator models; *c*’ refers to orientations’ direct effects on work satisfaction, when coping strategies are controlled. The indirect effect (*ab*) of orientations on work satisfaction through coping is represented by the product of pathways *a* and *b*. The total effect *c* is defined as the sum (*c*’ + *ab*) of the direct and indirect effects. To test the statistical significance of indirect effects, we implemented bootstrap confidence intervals. Bootstrapping is a resampling method generating an estimation of the sampling distribution of a statistic from the observed dataset. We used 10,000 bootstrap samples to compute bias-corrected bootstrap confidence intervals (95% CI). Point estimates of the indirect effects were considered to be significant when zero was not included in the 95% confidence intervals. Our sample size met the guidelines to reliably detect mediation effects by using bootstrapping (see Fritz and MacKinnon, 2007). Assuming small to medium effect sizes for both paths of mediation while determining a power of 0.80 (Cohen, 1988), the sample size required is between 71 and 391. In line with Fritz and MacKinnon (2007), we attempted to assess the maximum sample size.

## Results

### Preliminary Analyses

Means, standard deviations, and correlation analyses are shown in the electronic Supplementary Material (Supplementary Table S1; see footnote 1). There were small (*r* = −0.11–0.24) associations of the tested variables with age and/or gender. Therefore, we decided to control for these variables in the analyses conducted thereafter. The convergence between the self- and peer-rated orientations to happiness (self-other-agreement) were numerically lower for pleasure (*r* = 0.34) and engagement (*r* = 0.33) compared to meaning (*r* = 0.48), but in the same direction and in the expected range (cf. Ruch et al., 2010).

### Testing the Association Between Self- and Peer-Rated Orientations to Happiness, Work Satisfaction, Work Stress, and Stress Coping

Table 1 displays the correlations (controlled for age and gender) between self- and peer-reported orientations to happiness, types of work satisfaction, and work stress. All three self-rated orientations to happiness were positively associated with general *work satisfaction* and its content-related aspects. The overlap with the resigned aspects of work was considerably smaller (5% shared variance with the orientations to happiness, but 13 and 16% shared variance for general and content-related work satisfaction, respectively). The analysis of the peer-ratings showed that the ratings by knowledgeable others for engagement and meaning were also associated with content-related aspects of work satisfaction and that those were potentially the most robust relations. Overall, peer-rated pleasure seemed to be less predictive for types of work satisfaction.

**TABLE 1:** Correlations (controlled for age and gender) between self- and peer-reported orientations to happiness, types of work satisfaction, and work stress.

	Pleasure		Engagement		Meaning	
Self-reports	Self	Peer	Self	Peer	Self	Peer
*Work satisfaction*						
General	0.16	0.03	0.37***	0.11	0.30***	0.19*
Content-related	0.18*	0.09	0.40***	0.23*	0.30***	0.29**
Resigned	0.12	0.08	0.11	–0.07	0.21*	0.24***
*Work stress*						
General Work Stress	−0.21***	−0.21*	−0.18*	−0.23*	–0.15	–0.16

*N* = 369 for self-reported data, *N* = 100 for peer-self reported data. Types of work satisfaction values can range from 1 to 7, and orientations to happiness and work stress can range from 1 to 5 with higher scores indicating stronger endorsement. *p < 0.05. **p < 0.01. ***p < 0.001.

We found a negative relationship between the life of pleasure and experiencing *work stress*—numerically about the same size for peer-rated engagement. The experience of work stress further negatively correlated with the different types of work satisfaction (*r* = −0.42 to −0.58; not shown in the table).

We conducted hierarchical stepwise regression analyses with work satisfaction (general, content-related, resigned) and work stress as criteria (four separate analyses) and the three orientations to happiness as joint predictors (Step 2; see Table 2). Additionally, we controlled for the impact of age and gender in Step 1. The findings suggest that the orientations to happiness predicted the types of work satisfaction differently. *Post hoc* power analyses supported our proposed models (method: special increase in *R*^2^, fixed model, α = 0.05; Faul et al., 2009). In the final models, engagement predicted general work satisfaction (*f*^2^ = 0.15; 1 *−β* = 0.99), engagement and meaning predicted content-related work satisfaction (*f*^2^ = 0.19; 1 − β = 1.00), and meaning predicted resigned work satisfaction (*f*^2^ = 0.05; 1 *−β* = 0.97). The orientations to happiness shared 13 and 16% variance with general and content-related work satisfaction, respectively. Their predictive power for the resigned type was considerably smaller (5% shared variance). Overall, our findings suggest that work satisfaction was higher in those participants that endorsed engagement and meaning. Considering work stress, the dominant predictor among the orientation to happiness was the pleasure orientation (*f*^2^ = 0.06; 1 *−β* = 0.99), whereas the contributions of engagement and meaning were rather small in size and not significant. Hence, the overall amount of explained variance by the orientations was rather small (6% shared variance). The data met the assumption of collinearity, and multicollinearity was not a concern (median tolerance across all variables = 0.79 and median VIF = 1.26; see Supplementary Table S2 for details; see footnote 1).

**TABLE 2:** Hierarchical multiple regression analyses predicting types of work satisfaction (general, content-related, resigned) and work stress from age, gender, and orientations to happiness (pleasure, engagement, meaning).

	Types of work satisfaction			Work stress
	General	Content-related	Resigned	
Model	β	β	β	β
Step 1				
Age	0.13*	0.18***	0.12*	−0.08
Gender	−0.02	0.02	0.01	−0.09
Step 2: *Orientations to Happiness*				
Pleasure	0.05	0.01	0.08	−0.23***
Engagement	0.29***	0.30***	0.09	−0.06
Meaning	0.10	0.16**	0.11*	0.04
Δ*R^2^/R^2^*	0.13/0.16	0.16/.22	0.05/0.07	0.06/0.07
*F*	13.91***	19.98***	5.27***	5.18***

*N* = 370–372. Δ*R*^2^ = unique amount of explained variance by orientations of happiness. Types of work satisfaction values can range from 1 to 7 and orientations to happiness and work stress values can range from 1 to 5, with higher scores indicating endorsement. Age in years, gender: 1 = female, 2 = male. *p < 0.05. **p < 0.01. ***p < 0.001.

At the level of bivariate correlations (controlled for age and gender), the self-ratings of the life of pleasure increased with positive coping strategies and decreased with the total score of the negative strategies^2^ (see Table 3). Moreover, the life of engagement positively correlated with the total score out of the positive coping strategies and the global scores for the single facets, especially regarding the distraction and control strategies, and negatively with the total score out of the negative strategies. The pattern was similar for the life of meaning, while the strongest associations were discovered for the strategies targeting control. The life of meaning existed relatively independently from the total score of negative strategies. The findings also reveal that there existed between 3 and 20% shared variance connecting the self-reported three orientations to happiness and the stress coping strategies.

**TABLE 3:** Correlations between self- and peer-reported orientations to happiness and positive and negative coping strategies (controlled for age and gender).

	Pleasure		Engagement		Meaning		Δ*R*^2^	
				
Self-reports	Self	Peer	Self	Peer	Self	Peer	Self	Peer
**Positive Coping**	0.37***	0.22*	0.32***	–0.01	0.33***	0.20*	0.20	0.15
Devaluation	0.28***	0.19*	0.17**	0.03	0.16**	0.10	0.08	0.04
Minimization	0.23***	0.13	0.08	–0.07	0.17**	–0.02	0.07	0.03
Downplay	0.25***	0.15	0.24***	0.11	0.11**	0.16	0.09	0.04
Denial of guilt	0.15**	0.12	0.04	0.04	0.11*	0.16	0.03	0.02
Distraction	0.34***	0.24**	0.28***	0.01	0.30***	0.24**	0.17	0.11
Distraction	0.23***	0.10	0.21***	–0.05	0.14**	0.10	0.07	0.03
Substitute Gratification	0.23***	0.14	0.09	–0.05	0.16**	0.13	0.06	0.05
Self-affirmation	0.34***	0.24**	0.29***	0.07	0.23***	0.17*	0.15	0.08
Relaxation	0.24***	0.20*	0.27***	0.04	0.35***	0.26**	0.14	0.09
Control	0.25***	0.08	0.32***	–0.07	0.30***	0.11	0.14	0.03
Situation Control	0.15**	0.05	0.27***	0.05	0.23***	0.09	0.08	0.00
Reaction Control	0.12*	–0.04	0.17**	−0.22**	0.23***	0.05	0.06	0.07
Positive self-instructions	0.31***	0.18*	0.31***	0.01	0.25***	0.13	0.15	0.04
**Negative Coping**	−0.26***	−0.20*	−0.20***	–0.12	–0.08	–0.11	0.08	0.04
Escape	−0.21***	–0.03	−0.21***	–0.08	−0.13**	–0.03	0.06	0.00
Social withdrawal	−0.22***	–0.08	−0.12*	0.05	−0.11*	–0.01	0.05	0.01
Intrusive thoughts	−0.20***	–0.14	−0.17**	–0.08	–0.03	–0.08	0.06	0.03
Resignation	−0.26***	–0.16	−0.23***	–0.12	−0.10*	–0.10	0.09	0.03
Self-pity	−0.14**	−0.29**	−0.12**	−0.17*	–0.02	−0.17*	0.03	0.10
Self-blame	−0.25***	−0.23*	−0.13**	−0.20*	0.00	–0.16	0.06	0.07

N = 369 for self-reported data. N = 100 for peer-self reported data. ΔR^2^ = unique amount of explained variance by orientations of happiness when controlling for age and gender of the target person. Negative and positive coping strategy values can range from 0 to 4, and for pleasure, engagement, and meaning values can range from 1 to 5. *p < 0.05. **p < 0.01. ***p < 0.001.

If the peer ratings were taken into account, findings were comparable for the pleasurable life. As expected, the size of the correlation coefficients was smaller, but, again, the life of pleasure was associated with the positive strategies—in particular with those targeting distraction. The peer ratings for engagement were widely unrelated to self-rated coping strategies, while the peer-rated meaning was primarily associated with distraction (in particular, relaxation and self-affirmation). There was between 0 and 15% shared variance between the peer-rated orientations to happiness and the self-rated stress coping strategies. There was no association of the negative coping strategies with peer-reported orientations to happiness, except from the negative relationships between self-pity and peer-rated pleasure.

The data from self- and peer reports supported the notion that the three orientations to happiness contribute to both work-related well-being (work satisfaction, work stress) and coping with stress in the workplace. The findings show that the positive coping strategies (devaluation, distraction, and control) and the negative coping strategies were robustly related to the orientations to happiness.

### Testing a Mediation Model on the Contribution of Coping Strategies to the Association Between Orientations to Happiness and Well-Being at Work

We conducted separate mediation analyses for each type of work satisfaction and work stress using one of the three orientations to happiness as predictors (those revealed by the hierarchical regression analyses as the strongest predictor, namely, the engaged life for general and content-related work satisfaction, the meaningful life for resigned work satisfaction, and the pleasure life for work stress). We considered age and gender as covariates in all our analyses. Given that our mediators yielded robust bivariate correlations (e.g., *r* = 0.01–0.49), we computed mediation analyses with parallel mediators to simultaneously test the contribution of devaluation, distraction, control (positive coping), and negative coping in mediating the effects of orientations to happiness on work satisfaction and work stress.

**FIGURE 2:**
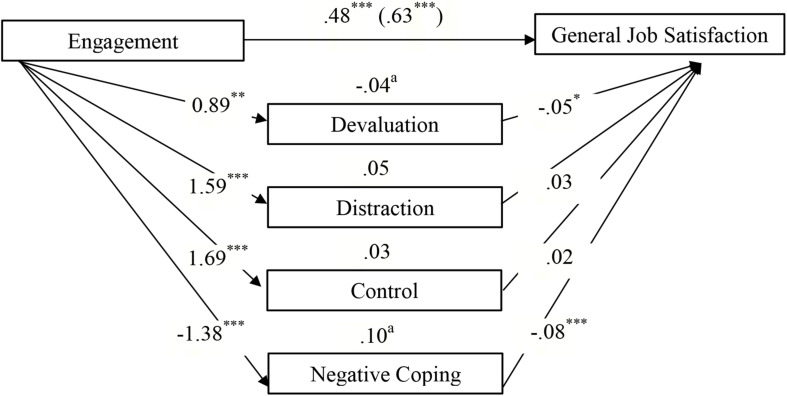
Direct, indirect, and total effects (in brackets) of Engagement on General Job Satisfaction via the mediators Positive (Devaluation, Distraction, Control) and Negative Coping Strategies (Controlled for age and gender). ^∗^*p* < 0.05. *^∗∗^p* < 0.01. *^∗∗∗^p* < 0.001. ^a^Significant point estimates (*p* < 0.05).

**FIGURE 3:**
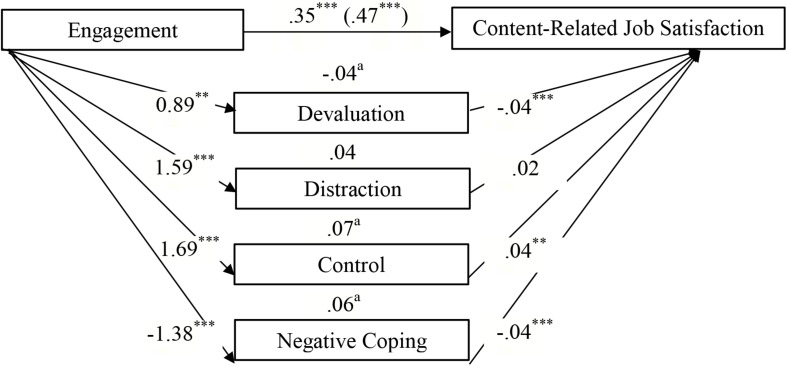
Direct, indirect, and total effects (in brackets) of Engagement on content-related Job Satisfaction via the mediators Positive (Devaluation, Distraction, Control) and Negative Coping Strategies. It was controlled for age and gender. *^∗∗^p* < 0.01. *^∗∗∗^p* < 0.001. ^a^Significant point estimates (*p* < 0.05).

**FIGURE 4:**
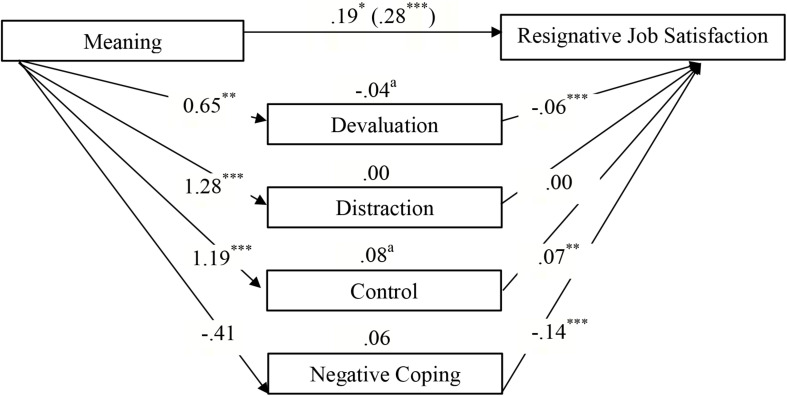
Direct, indirect, and total effects (in brackets) of Meaning on Resignative Job Satisfaction via the mediators Positive (Devaluation, Distraction, Control) and Negative Coping Strategies (Controlled for age and gender). ^∗^*p* < 0.05. *^∗∗^p* < 0.01. *^∗∗∗^p* < 0.001. ^a^Significant point estimates (*p* < 0.05).

Table 4 shows that the effect of the engaged life on *general work satisfaction* was partially mediated by devaluation and negative coping, whereas no mediation effect was found for distraction and control (Figure 2). Unexpectedly, there was a *negative* effect of devaluation on work satisfaction. A comparison of indirect effects (devaluation minus negative coping) revealed a specific contrast effect of −0.15 (*SE* = 0.04, 95% CI = −0.24 to −0.07). The inspection of the bootstrap confidence interval shows that it excluded zero; hence, the effects of negative coping were significantly stronger than the effects for devaluation. Furthermore, parts of the link between the engaged life and *content-related work satisfaction* were partly transmitted via devaluation, control, and negative coping, whereas no mediating effect was found for distraction (Figure 3). Comparing indirect effects revealed specific significant contrasts for devaluation minus control (−0.11, *SE* = 0.04, 95% CI = −0.19 to −0.05) and devaluation minus negative coping (−10, *SE* = 0.03, 95% CI = −0.17 to −0.05), thereby indicating that devaluation yielded a smaller indirect effect than control and negative coping, while there was no significant difference among the latter (0.01, *SE* = 0.03, 95% CI = −0.05 to 0.08). The association between the meaningful life and *resigned work satisfaction* was partly mediated by positive coping strategies, that is, devaluation and control, whereas no indirect effect was identified for negative coping (Figure 4). Among the positive coping strategies, the mediating effect of control was stronger than that of devaluation (−0.12, *SE* = 0.04, 95% CI = −0.20 to −0.05). Our findings show that the effects of engagement in general or content-related work satisfaction were mediated mainly by negative coping and control, while the association between meaning and resigned work satisfaction was mediated by positive coping.

**TABLE 4:** Parallel mediation analyses predicting types of work satisfaction and work stress (DV) (10,000 bootstrap samples).

	Independent variable (IV)	Mediating variable (M)	Dependent Variable (DV)	Effect of IV on M (*a*)	Effect of M on DV (*b*)	Direct effect (*c*’)	Total effect (*c*)	Indirect effect	
								(*ab*)	95% CI
1	Engagement	Devaluation	General	0.89**	−0.05*	0.48***	0.63***	−0.04^a^	(−0.09;−0.01)
		Distraction		1.59***	0.03			0.005	(−0.01;0.12)
		Control		1.69***	0.02			0.03	(−0.03;0.11)
		Negative Coping		−1.38***	−0.08***			0.10^a^	(0.05;0.17)
2	Engagement	Devaluation	Content-related	0.89**	−0.04***	0.35***	0.47***	−0.04^a^	(−0.08;−0.01)
		Distraction		1.59***	0.02			0.04	(0.00;0.09)
		Control		1.69***	0.04**			0.07^a^	(0.03;0.12)
		Negative Coping		−1.38***	−0.04***			0.06^a^	(0.02;0.10)
3	Meaning	Devaluation	Resigned	0.65**	−0.06***	0.19***	0.28***	−0.04^a^	(−0.06;−0.01)
		Distraction		1.28***	–0.00			0.00	(−0.06;0.06)
		Control		1.19***	0.07**			0.08^a^	(0.02;0.15)
		Negative Coping		–0.41	−0.14***			0.06	(−0.02;0.14)
4	Pleasure	Devaluation	Work stress	1.39***	–0.02	–0.09	−0.26***	–0.02	(−0.05;0.01)
		Distraction		1.79***	0.02			0.03	(−0.01;0.07)
		Control		1.20***	–0.01			–0.01	(−0.04;0.01)
		Negative Coping		−1.67***	0.10***			−0.16^a^	(−0.24;−0.10)

*N* = 370. All mediation analyses were conducted while controlling for gender and age. Negative and positive coping strategy values can range from 0 to 4, and for pleasure, engagement, meaning, and work stress values can range from 1 to 5, and types of work satisfaction values can range from 1 to 7 with higher scores indicating endorsement. Parameter estimates are unstandardized regression coefficients. *p < 0.05. **p < 0.01. ***p < 0.001. ^*a*^Significant point estimates (*p* < 0.05) using 95% Bootstrapping confidence interval (95% CI).

**FIGURE 5:**
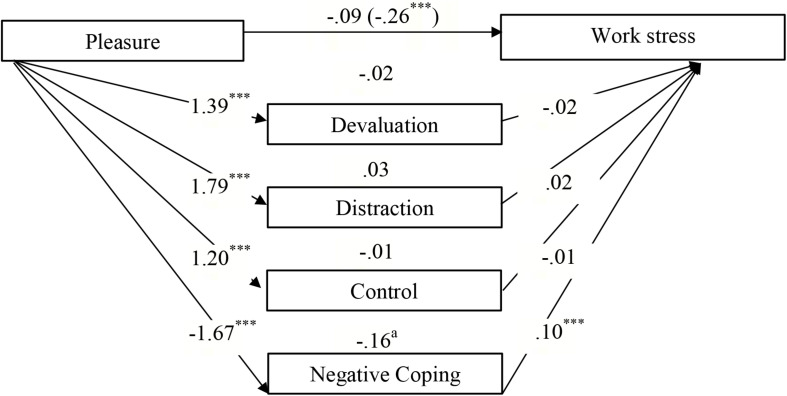
Direct, indirect, and total effects (in brackets) of Pleasure on Work Stress via the mediators Positive (Devaluation, Distraction, Control) and Negative Coping Strategies (Controlled for age and gender).*^∗∗∗^p* < 0.001. ^a^Significant point estimates (*p* < 0.05).

The association between the orientation to pleasure and reported *work stress* was wholly transmitted via negative coping, whereas the devaluation, distraction, and control positive coping strategies did not contribute significantly to the mediation (Figure 5).

## Discussion

The main aim of the present study was to extend earlier findings on the relationship between orientations to happiness and work-related well-being in a sample of Swiss employees by including peer ratings of the three orientations to happiness. Overall, we found that the three orientations relate to higher levels of satisfaction with one’s working life, in particular, the engaged life (for facets, also the life of meaning). Additionally, the pleasurable life may be vital in experiencing lower stress levels at the workplace. However, the causality is unclear, and higher well-being at work may relate to higher orientations to happiness.

### Orientations to Happiness and Well-Being at Work

The life of engagement emerged as a potent predictor of *general work satisfaction*. Engagement and meaning predicted *content-related work satisfaction* and meaning predicted *resigned work satisfaction*. However, the association for the resigned type was considerably smaller than for the other types. Greater self-rated endorsement of a pleasurable life was associated with lower experiences of *work stress*, which may point to potential buffering effects. The analyses based on the peer ratings support the importance of engagement and meaning for content-related work satisfaction.

Our results are comparable to earlier research (Proyer et al., 2012; Swart and Rothmann, 2012; Martínez-Martí and Ruch, 2017), which emphasized the importance of engagement for work satisfaction. This is also in line with Seligman (2002), who proposed that individuals usually feel more engaged at work than at home (see Ruch et al., 2010, 2014). The experience of flow, which is related to engagement, seems, therefore, to occur more frequently at work than during leisure time (Csikszentmihalyi and LeFevre, 1989) and may hold the potential to elicit positive emotions and contribute to well-being. Of course, our data do not allow for the interpretation of causal effects. Future research may test whether workplace interventions directed at improving an engaged (and meaningful—and also a pleasurable) life hold the potential to increase positive experiences and decrease negative emotions at work and the elicitation of positive emotions. Finally, the inclusion of other outcomes (e.g., integrity, commitment, or innovative behavior at work) would be helpful in obtaining a better understanding of the role of the orientations at work. Earlier research has shown that all three orientations to happiness relate positively to different indicators of well-being (correlational, for example, Peterson et al., 2007; Ruch et al., 2010; experimental, for example, Giannopoulos and Vella-Brodrick, 2011; Gander et al., 2016, 2017; Proyer et al., 2016), and our study supports the notion that they also have an impact on well-being at work. More research on work-related interventions is needed to determine how employees can capitalize from their orientations to happiness for a better experience at work.

### Orientations to Happiness and Coping Strategies

We further considered positive coping strategies such as devaluation, distraction, and control and the single dimension for negative coping to gain more insight into the possible regulating mechanisms that help to implement the adaptive associations of the orientations to happiness to experience well-being in work life.

The *life of pleasure* was related to all three subscales of the positive coping strategies (devaluation, distraction, and control) and to lower expressions in the negative coping strategies. As expected, the peer ratings were in the same direction—yet lower in size. This supports the notion that pleasure plays an important role in coping with work stress. One might argue that people scoring high in life of pleasure find it easier to distract themselves from problems and stressful experiences and devalue them to cope with adversity. They have a good understanding of what activities elicit flow experiences, enabling them to do things that are incongruous to what causes the stressful experience. Simultaneously, they avoid or resign (flight) to a lower degree, both of which are negative coping strategies that do not help one to cope with the problem. This helps one to cope with the acute intensity of the problem and offers diversion but allows people to attend the problem later (after having distracted themselves to recuperate). A further assumption is that these people generally take life a little “easier” by trivializing difficulties and not taking them too seriously—thinking that they will be able to cope with what comes their way and also be more open to improvise given the circumstances. One consequence of this might be that they experience less stress at work. Whether this should be seen as a protective factor—only preventing stress from occurring—or whether too much endorsement of a pleasurable life also pertains to certain risks for other outcomes (e.g., integrity, involvement in work tasks, or productivity) may be valuable research questions for future research relying on longitudinal designs.

The *life of engagement* yielded associations with the positive coping strategies of devaluation and distraction and, specifically, control, and with the negative coping strategy. These results are in line with our expectations of flow as a state of total absorption (i.e., distraction from other things) with a strong feeling of having things under control. Overall, these findings warrant replication as the analysis of the peer ratings did not support the findings.

Self-ratings of the *meaningful life* were also associated with the positive coping strategies of distraction and, in particular, control. The peer ratings for meaning were primarily associated with distraction. A life of meaning is associated with the attitude that life serves a higher purpose. A connection between this attitude and control strategies seems reasonable. The strategy “situation control” is, for example, about analyzing situations and planning actions for control/problem solving. Hence, those high in a life of meaning may actively try to make something positive out of a given problem and learn from it for the benefit of a higher goal. Simultaneously, striving for meaning in life is unrelated to negative coping strategies such as ruminating about events, blaming oneself, and feeling sorry for oneself. Striving for a higher meaning in life can be understood as part of the intrinsic motive system (Elliot and Church, 1997), which can be diminished by external punishment, whereas the meaning orientation seems to be unsusceptible to negative thoughts about the self or to ruminating.

In summary, we propose that the orientations to happiness, especially pleasure, may function as personal resources and contribute to more positive and less negative coping. As previously mentioned, we cannot make statements on causality. The reason that we found slightly different results for the peer ratings (e.g., no associations between peer-rated engagement and coping) could be due to the fact that personality traits and behaviors differ in their observability/evaluativeness (see Vazire, 2010). For example, one might argue that flow experiences are more difficult to recognize in others than pleasure or meaning. Furthermore, the ratings of the acquaintances likely do not refer explicitly to the individual’s behavior at work, since the majority of them were friends (or the partner/spouse) from their private life, not their work life. Moreover, it needs to be taken into account that peer ratings only exist for a subset of all participants providing self-ratings and that the peer ratings have, therefore, less stability in comparison with the larger sample of self-ratings (e.g., when taking research on the stability of correlation coefficients into account; Schönbrodt and Perugini, 2013).

### The Relationship of Orientations to Happiness and Coping Strategies With Well-Being at Work

We proposed that the positive and negative coping strategies would mediate the associations between all the happiness orientations and well-being at work (facets of work satisfaction and work stress). Overall, reporting higher engagement at the workplace is related to experiencing higher levels of *general work satisfaction*, whereas this association is partly transmitted via the positive coping strategy devaluation of stressful experience and less negative coping, whereas negative coping was the predominant mediator. Considered in light of the transactional model of stress (Lazarus and Folkman, 1984a, b), being wholly absorbed in current tasks increases general work-related satisfaction because engagement enables people to not avoid or resign (flight) from difficulties that do not help one to cope with the problem. Comparably, the positive association of engagement in the workplace with reporting experiencing *content-related work satisfaction* is partly mediated by the positive strategies of devaluation and control, and less by negative coping strategies, whereas the mediating effects of control and negative coping were dominant over devaluation. Besides the aforementioned mediating mechanisms of negative coping, control seems to be important in mediating this effect. Arguably, because control helps to analyze situations and plan actions to control and solve problems related to work content, control accompanies increased content-related work satisfaction. The positive association between experiencing meaning at work and *resigned work satisfaction* is partly transmitted via the devaluation of stressors and active control of the stressors, whereas again, control was the predominant mediator. Our results suggest that striving for a life of meaning is associated with higher levels of actively trying to make something positive out of a given situation, which means more resigned work-related satisfaction.

Given only the partial mediation effects, there are still effects of engagement and meaning on work satisfaction that are independent of our considered coping mechanisms. Thus, it seems fruitful to continue studying potential mediators. One moderating variable of these effects may be playfulness [i.e., individual differences in the propensity to play and to (re)frame everyday situations in such a way as to experience entertainment, and/or intellectual stimulation, and/or personal interest; Proyer, 2017]. One might argue that a group of adults in the workplace may seek pleasure (at work) from playful interactions with others, intellectual types of challenges, or a general tendency toward lightheartedness and seeing problems from a meta-perspective enabling them to better cope with problems. Potential support for this notion comes from research showing that there is a robust relationship between playfulness and (primarily) a life of pleasure and an engaged life (Proyer et al., 2012; Proyer, 2014) and that playfulness moderates the relationship between behaviors related to a playful work design and both work engagement and creativity (Scharp et al., 2019).

Considering the association between the pleasure orientation and experienced *work stress*, we found that the negative coping strategy functions as a total and unique mediator even in the presence of the devaluation, distraction, and control positive coping strategies. Less application of negative coping strategies such as self-pity, self-blame, or rumination seems to serve as a mechanism through which people attain the maximization of pleasure and the minimization of pain at work. This interpretation is also consistent with our framework of the transactional model of stress (Lazarus and Folkman, 1984a, b). In the present situation, our negative coping strategies can be categorized as emotion-focused coping. Emotion-focused coping relates to the inner processes of individuals and helps to reduce negative emotional states such as the given work-related stress or to change the appraisal of the demanding work-related stress but does not relate to actively planning or engaging in activities to overcome the problem causing distress.

#### Self-Other-Agreement in the Orientations to Happiness

Data collected in this study allow for a replication of the self-other-agreement in the three orientations to happiness (Ruch et al., 2010). As expected, self- and peer ratings converged well (between *r* = 0.33 and 0.47). However, they were numerically lower for pleasure and engagement than in Ruch et al. (2010). There may be differences in the level of acquaintanceship, methodology (two raters in Ruch et al. (2010), while we only had one), and sample sizes that may help explaining these findings. In any case, self-other-agreement in the orientations to happiness is in a comparable range to what has been reported in the literature for trait measures.

Taking our findings together, we argue for a stronger consideration of orientations to happiness in their relationship with work-related variables. The study extends prior knowledge in the field (e.g., Proyer et al., 2012; Swart and Rothmann, 2012; Johnston et al., 2013) and shows their potential for dealing with work-related stress.

### Limitations

Our results are based on a cross-sectional design. We, therefore, cannot make any comments about causality or the directionality of the relationship between orientations to happiness, work satisfaction, work stress, and coping strategies. Based on our theoretical model (transactional stress model; Lazarus and Folkman, 1984a, b), we argue that the experiencing of the orientations to happiness may facilitate certain coping strategies and behaviors and eventually more well-being at work, but it is also possible that the effect is vice versa—or based on interactions or the effects of other variables (e.g., employment status; Gander et al., 2019). A longitudinal research design should be applied in future research. Alternatively, the assumed causal link from the orientations to happiness on well-being via coping strategies can also be proofed using an experimental design such as the “experimental-causal-chain-design” (Spencer et al., 2005) or the testing-a-process-hypothesis-by-an-interaction-strategy-Ansatz (Jacoby and Sassenberg, 2011).

Given that we tried to overcome the common method bias by not relying on self-reports alone, we additionally included peer rating of the happiness orientations. However, with respect to the peer ratings of the orientations to happiness, the “letter of recommendation” effect could play a role: informants were nominated by targets, mostly friends and partners, and such informants may tend to like the target; thus they describe them overly positively (Leising et al., 2010). Hence, a follow-up study with a different recruitment strategy or the inclusion of more knowledgeable persons, possibly from the work environment, providing the peer ratings should be considered—as well as having multiple peer raters. Furthermore, peer ratings were not available for the full sample, but only a subsample of those that have provided self-reports. Taking simulation studies of, for example, stability of estimates for correlation coefficients, into account (e.g., Schönbrodt and Perugini, 2013), the findings for the self-reports have more stability, and findings for the peer ratings need to be interpreted with more caution. Another strategy to overcome possible method biases would be using unmeasured method factor techniques as suggested by Podsakoff et al. (2012) or using structural equation models. However, given the cautionary note on the role of the peer ratings mentioned earlier, we suggest applying such analyses in a replication study with more balanced samples sizes across self- and peer ratings.

Our study demonstrated associations of orientations to happiness with facets of work-related well-being in a general sample of employees. Future research should build on this by validating our models in participants working in particular domains such as in less vs. highly skilled occupations. Previous research has already shown different associations between orientations to happiness and general life satisfaction across occupations (Hofmann et al., 2018).

Finally, Seligman (2011) has revised his initial model, which now covers five facets (the inclusion of the achieving life/Accomplishment and positive relationships^3^), and the pleasurable life is reconceptualized in the sense of a proneness to experience positive emotions. Only comparatively recently have measures been developed for the assessment of these additional components in German-speaking countries (English version: Butler and Kern, 2016; Japanese version: Watanabe et al., 2018; German version: Wammerl et al., 2019), and so we could not implement them in this study. Hence, a future replication and extension should be based on this conceptualization. Current research projects using the PERMA model demonstrate already promising results (e.g., Tansey et al., 2017; Wagner et al., 2020).

### Implications

Since the orientations to happiness relate to adaptive coping with work stress and work satisfaction, consideration should be given to the possibility of enhancing the orientations to happiness in the workplace (e.g., via positive psychology interventions). Indeed, Meyers et al. (2013) conducted a review of positive psychology interventions applied in the working field. They identified 15 studies that examined the effects of interventions that were based on the cultivation of positive subjective experiences, the building of positive individual traits, or the building of civic virtue and positive institutions. In doing so, they concluded that positive psychology interventions consistently enhance employee well-being and performance and tend to diminish stress and burnout and, to a lesser extent, depression and anxiety. With regard to the orientations to happiness, the life of engagement, respectively the experience of flow and the life of meaning, seems to be crucial for work satisfaction and may, therefore, be a good starting point for the development of further interventions. They could be enhanced if employees identify their strengths and have the possibility to use them at work (see also Seligman, 2002). Typically, positive psychology interventions can easily be integrated into daily routines, can be self-administered (e.g., in online settings), and do not take up too much time or use too many materials (e.g., Sin and Lyubomirsky, 2009). Interventions that focus on the implementation of positive coping strategies in the work field should be additionally considered, for example, training based on strengthening individuals’ intrapreneurial self-capital (e.g., Di Fabio, 2014) that is regarded as a set of individual resources that enables people to proactively cope with the uncertainties of the 21st century’s world of work.

In summary, intervention studies based on the three orientations are promising, and while it has already been shown that these interventions contribute to subjective well-being (Giannopoulos and Vella-Brodrick, 2011; Gander et al., 2016, 2017, 2018; Proyer et al., 2016), it seems reasonable to assume that they also hold potential for increasing work-related well-being (Harzer and Ruch, 2012, 2015).

## Conclusion

Overall, our results indicate that the orientations to happiness are of importance for work-related well-being, such as general, content-related, and resigned work satisfaction and experienced work stress. In particular, the life of pleasure seems to be helpful for coping with work stress, while the life of engagement and, to a lesser extent, the life of meaning contribute to work satisfaction. This finding is in line with our theoretical transactional stress model and the positive psychology framework. The orientations to happiness function as positive traits that foster positive subjective experience such as well-being in the workplace mainly via using positive coping strategies.

Future research may examine the possibility of enhancing the orientations to happiness in the workplace and studying the impact of these activities on different indicators of well-being at work. From our point of view, the occupational context may benefit if we started to study and eventually facilitated more aspects related to the field of Positive Psychology—aspects that help researchers to understand what adds meaning to work life and promotes optimal functioning (Seligman and Csikszentmihalyi, 2000).

## Data Availability Statement

All data are available on the Open Science Framework: https://osf.io/mf5cj/.

## Ethics Statement

The study was conducted in compliance with the local ethical guidelines at the University of Zurich (place of data collection) and participants provided online informed consent. Written informed consent for participation was not required for this study in accordance with the national legislation and the institutional requirements.

## Author Contributions

AK and RP contributed to the conception and the design of the study. AK organized the data analyses. NT conducted the statistical analyses. AK, NT, and RP wrote sections of the manuscript. All the authors engaged in revisiting, reading, and approving the submitted version of the manuscript.

## Conflict of Interest

The authors declare that the research was conducted in the absence of any commercial or financial relationships that could be construed as a potential conflict of interest.

## Footnotes

1 https://osf.io/mf5cj/

2 Due to the high number of simultaneous correlational tests, there is a risk of alpha-error accumulation. We, therefore, rejected the null hypotheses only when the *p*-value was lower than 0.01.

3 Seligman (2011) defines Accomplishment as “[…] success, accomplishment, winning, achievement, and mastery for their own sake” (p. 18) given that “[…] people often acted simply to exert mastery of the environment” (p. 20). Furthermore, he argues that “[…] other people are the best antidote to the downs of life and the single most reliable up” (p. 20) and stresses the importance of positive relationships with others (e.g., covering feelings of trust toward others and close relationships).

## Supplementary Material

TABLE S1 Means, standard deviations, and correlations of orientations to happiness (OTH) and well-being at work with gender and age.

TABLE S2 Collinearity analyses for our multiple regression models predicting types of work satisfaction (General, Content-related, Resigned) and work stress from age, gender, and orientations to happiness (Pleasure, Engagement, Meaning).
