# Supplementary material for: Authentic Happiness at Work: Self- and Peer-Rated Orientations to Happiness, Work Satisfaction, and Stress Coping
Source: Front Psychol. 2020 Aug 7;11:1931. doi: 10.3389/fpsyg.2020.01931 (PMC7426460; doi:10.3389/fpsyg.2020.01931)
Supplement: TABLE S1 — Means, standard deviations, and correlations of orientations to happiness (OTH) and well-being at work with gender and age. [file Data_Sheet_1.docx]

**Electronic supplementary material:**

Supplementary Table 1 - ESM A

*Means,Standard deviations. and Correlations of Orientations to Happiness (OTH) and Well-being at work with gender and age*

|  | |  | *M* | *SD* | α | *S* | *K* |  | Age | Gender |
| --- | --- | --- | --- | --- | --- | --- | --- | --- | --- | --- |
| Self-OTH | | |  |  |  |  |  |  |  |  |
|  | Pleasure | | 3.27 | 0.67 | .73 | -0.24 | -0.28 |  | -.12^*^ | -.19^***^ |
|  | Engagement | | 3.08 | 0.61 | .68 | -0.15 | -0.32 |  | .14^**^ | .02 |
|  | Meaning | | 3.08 | 0.82 | .79 | -0.08 | -0.31 |  | .11* | -.03 |
| Peer-OTH | | |  |  |  |  |  |  |  |  |
|  | Peer-Pleasure | | 3.34 | 0.68 | .75 | -0.13 | -0.35 |  | .01 | .01 |
|  | Peer-Engagement | | 3.33 | 0.61 | .64 | 0.00 | 0.07 |  | -.03 | -.10 |
|  | Peer-Meaning | | 3.38 | 0.79 | .80 | -0.31 | -0.83 |  | .02 | .07 |
| Job Diagnostic Survey | | |  |  |  |  |  |  |  |  |
|  | Resigned WS | | 5.41 | 1.31 | .81 | -0.65 | -0.36 |  | .14^**^ | .01 |
|  | Content-related WS | | 4.27 | 0.77 | .86 | -0.53 | -0.05 |  | .24^***^ | .04 |
|  | General WS | | 4.48 | 1.10 | .88 | -0.80 | 0.18 |  | .17^***^ | -.01 |
| General Work Stress Scale | | | 1.99 | 0.71 | .90 | 0.93 | 0.55 |  | -.06 | -.06 |
| Stressverarbeitungsfragebogen-120 | | |  |  |  |  |  |  |  |  |
|  | *Positive* *CS* | | 12.23 | 2.69 | .93 | -0.36 | 0.35 |  | .08 | -.15^**^ |
|  | *De Devaluation* | | 10.01 | 3.27 | .86 | -0.16 | 0.38 |  | .03 | -.07 |
|  | To trivialize | | 11.03 | 4.26 | .79 | -0.04 | 0.05 |  | -.04 | -.14^**^ |
|  | To deemphasize | | 10.17 | 4.71 | .85 | 0.26 | -0.13 |  | .07 | .09 |
|  | To deny guilt | | 8.81 | 3.83 | .79 | -0.03 | -0.28 |  | .04 | -.13^*^ |
|  | *Distraction* | | 11.40 | 3.48 | .90 | -0.16 | -0.03 |  | -.07 | -.16^**^ |
|  | Distraction | | 8.81 | 3.83 | .67 | -0.03 | -0.30 |  | .04 | -.13^*^ |
|  | Displacement activity | | 10.19 | 4.74 | .81 | 0.09 | -0.10 |  | -.05 | -.19^***^ |
|  | Validation of self | | 11.76 | 4.53 | .85 | -0.12 | -0.08 |  | .12^*^ | -.09 |
|  | Relaxation | | 11.72 | 5.41 | .89 | 0.03 | -0.47 |  | .18^***^ | -.11^*^ |
|  | *Control* |  | 15.56 | 3.24 | .87 | -0.34 | 0.31 |  | .08 | -.11^*^ |
|  | Control of situation | | 16.77 | 3.98 | .82 | -0.24 | -0.44 |  | .18^***^ | -.04 |
|  | Control of reaction | | 14.01 | 3.96 | .73 | -0.24 | 0.23 |  | -.04 | -.15^**^ |
|  | Positive self-instruction | | 15.90 | 4.40 | .85 | -0.63 | 0.61 |  | .05 | -.06 |
|  | *Negative* *CS* | | 8.96 | 4.22 | .96 | 0.40 | -0.19 |  | -.20^***^ | -.17^**^ |
|  | Flight | | 7.76 | 5.52 | .90 | 0.70 | 0.02 |  | -.17^***^ | -.15^**^ |
|  | Social withdrawal | | 6.95 | 5.21 | .91 | 0.76 | -0.01 |  | -.14^**^ | -.05 |
|  | Mental occupation with stressor | | 13.81 | 5.80 | .93 | -0.06 | -0.67 |  | -.15^**^ | -.16^**^ |
|  | Resignation | | 6.94 | 4.71 | .87 | 0.64 | 0.02 |  | -.20^***^ | -.16^**^ |
|  | Self-pity | | 7.92 | 5.00 | .86 | 0.58 | -0.20 |  | -.15^**^ | -.12^*^ |
|  | Self-accusation | | 10.37 | 4.98 | .86 | 0.37 | -0.05 |  | -.16^**^ | -.16^**^ |

*Notes. N =* 368 – 372/100. Gender: 1= female. 2 = male. *S* = Skewness; *K* = Kurtosis; α = Cronbach-Alpha. WS = Work satisfaction; CS = Coping strategies.

^*^*p* < .05. ^**^*p* < .01. ^***^*p* < .001.

| Supplementary Table 2 - ESM B  *Collinearity analyses for our Multiple Regression Models Predicting Types of Work Satisfaction (General, Content-related, Resigned) and Work Stress from Age, Gender, and Orientations to Happiness (Pleasure, Engagement, Meaning)* | | | | | | | | | | | | | |
| --- | --- | --- | --- | --- | --- | --- | --- | --- | --- | --- | --- | --- | --- |
|  | Types of Work Satisfaction | | | | | | | |  | Work Stress | | | |
| Model | General | |  | Content-related | |  | Resigned | |  |  | | | |
|  | Tolerance | VIF |  | Tolerance | VIF |  | Tolerance | VIF |  | Tolerance | | | VIF |
| Step 1: *Demographics* |  | |  |  | |  |  | |  |  | | | |
| Age | .94 | 1.06 |  | .94 | 1.06 |  | .94 | 1.06 |  | .94 | | 1.06 | |
| Gender | .95 | 1.05 |  | .95 | 1.05 |  | .95 | 1.05 |  | .95 | | 1.05 | |
| Step 2: *Orientations to Happiness* | | |  |  | |  |  | |  |  | | | |
| Pleasure | .79 | 1.26 |  | .79 | 1.26 |  | .79 | 1.26 |  | .79 | 1.26 | | |
| Engagement | .73 | 1.38 |  | .73 | 1.38 |  | .73 | 1.38 |  | .73 | 1.38 | | |
| Meaning | .79 | 1.27 |  | .79 | 1.27 |  | .79 | 1.27 |  | .79 | 1.27 | | |
| Cook’s distance (*M*, *SD*) | .003, .005 | |  | .003, .005 | |  | .003, .004 | |  | .003, .005 | | | |
| *Note*. *N* = 370-372. VIF = variance inflation factor. | | | | | | | | | | | | | |
